# Strengthening NVVC endorsements for ESC guidelines

**DOI:** 10.1007/s12471-024-01905-4

**Published:** 2024-09-19

**Authors:** Pim van der Harst

**Affiliations:** Department of Cardiology, Division of Heart and Lungs, University Medical Centre Utrecht, Utrecht, The Netherlands

Cardiology is a constantly evolving field, and with that comes the responsibility of ensuring that international guidelines and recommendations, such as those from the European Society of Cardiology (ESC), are adapted to fit the national context of the Netherlands. To meet this challenge, the Netherlands Society of Cardiology (NVVC) has taken significant steps to formalize its endorsement process. In this issue, we begin with the endorsement of the ESC guidelines for acute coronary syndromes (ACS) [1].

This marks the launch of a structured protocol that clearly divides the tasks and responsibilities between the NVVC and the Editorial Board of the Netherlands Heart Journal (NHJ). Our goal is to maintain consistency, transparency, and relevance while delivering practical, actionable guidance for the cardiology community in the Netherlands.

The process begins by selecting the most relevant ESC guidelines to endorse for the national context. The NVVC then assembles a writing group of experts to draft the endorsement. Each document undergoes an open internal review by NVVC members and an external review by independent experts. All participants, including reviewers, are required to declare the ‘NVVC conflict of interest statement’ before the endorsement is approved by the NVVC subcommittee on Guidelines of the Quality Committee and the NVVC Board to be submitted to the NHJ. Additionally, the final version of the document will be approved by the NVVC Board before publication.

Our aim is to continue providing high-quality, well-considered endorsements that support and improve cardiology practice in the Netherlands. This same framework will also apply to future NVVC publications, including position statements, consensus documents, and recommendations. The NVVC and NHJ will work closely together to assess and improve this process as we move forward. We are proud of this advancement and look forward to sharing future endorsements and official NVVC publications.
